# Nutrient dataset development via FAO/INFOODS approach for infant nutritional survey in rural Matiari, Pakistan

**DOI:** 10.1016/j.jfca.2024.106471

**Published:** 2024-09

**Authors:** Sanam Iram Soomro, Zehra Jamil, Najma Memon, Sheraz Ahmed, Fayaz Umrani, Imran Ahmed Choudhri, Sajid Mohammed, Khalique Qureshi, Ghulam Raza, Sadaf Jakhro, Asad Ali

**Affiliations:** a Department of Community Health Sciences, Aga Khan University, Karachi, Pakistan; b Biological & Biomedical Sciences, Aga Khan University, Karachi, Pakistan; c National Centre of Excellence in Analytical Chemistry, University of Sindh, Jamshoro, Pakistan; d Division of Woman and Child Health, Aga Khan University, Karachi, Pakistan; e Department of Pediatrics and Child Health, Aga Khan University, Karachi, Pakistan

**Keywords:** Malnutrition, Food composition data, Infants, Stunting

## Abstract

To accurately evaluate dietary intake, multiple resources are necessary, including serving-size modules, pictures, and questionnaires that are used to gather information during surveys. One critical component is the accessibility of food composition data at the national or regional level, which is required to determine dietary intake. Food Agriculture Organization/International Network of Food Data Systems (FAO/INFOODs) tools are useful for developing high-quality food composition data. We used these tools to create a nutrient dataset for a nutritional survey in Matiari, Sindh, and to collect dietary information through a 24-hour food recall questionnaire. The survey results indicated 540 distinct types of foods, including 291 ready-to-eat items, 84 foods used as ingredients in recipes, and 164 various composite and mixed recipes. Most food items corresponded to the national and regional Food Composition Tables (FCTs) and the Food and Nutrient Database for Dietary Studies (FNDDS) of the USDA, with the exception of recipe food data. We utilized Eurofir-recipe calculation methods to compute the recipe data. The data were homogenized and standardized utilizing EFSA and Langual™. Because of the obsolescence and inadequacy of Pakistan's food composition table in assessing essential nutrients, we had to source data from various other sources. Consequently, to establish the nutrient dataset, we incorporated approximately 25 % of user data from national sources, with recipe data comprising 46 % and less than 20 % extracted from regional, U.S database, and diverse online sources. This study is the first effort in which we gathered data from reliable sources representing local eating patterns, with some exceptions. Future studies will hugely benefit from this database, especially as we face a high prevalence of undernutrition in our part of the world.

## Highlights

• Food composition databases updated for Pakistani children with high stunting rates.
• South Asian recipes are matched using reliable databases like Food Explorer Interface.
• EuroFIR calculated 162 unmatched recipes using regional food composition databases.
• Cost-effective stepwise methodology for LMIC researchers to develop nutrient datasets.

## 1 Introduction

The 24-hour dietary recall (24hDR) is a commonly used method in nutrition surveys worldwide. It is frequently combined with other methods such as food frequency questionnaires, diet records, and diet history. The 24hDR has also been utilized in various editions of the National Health and Nutrition Examination Study. The 24-hour dietary recall (24hDR) is a subjective, retrospective method that can be administered through face-to-face or telephone interviews or self-administered via computer-based programs and web-based online systems. This method involves accurate recall, description, and quantification of all food forms cooked, uncooked, or processed consumed in the previous 24 h, from the first intake in the morning to the last before bed or any midnight snacks. (Castell et al. 2015; Thompson and Subar, 2017). The 24-hour recall method has several advantages. Unlike self-administered versions, it can record responses without requiring respondent literacy. The recall period allows participants to remember most of their dietary intake, making this method more representative of the population than those who maintain food records. Trained interviewers can capture necessary details for accurate food coding. In addition, because dietary recalls are conducted after food consumption, reactivity is less of an issue. However, the main drawback is that individuals may not accurately report their food consumption owing to knowledge, memory, and interview conditions. Multiple days of recall may be necessary to meet the objectives of the study. While a single 24-hour recall can describe the average dietary intake of a population, multiple days are required to accurately model the usual intake distributions. Repeated 24-hour recalls also allow for a more precise estimation of relationships with other factors. In recent years, digital technologies have revolutionized dietary data collection, enabling the use of web-based mobile apps and image-capturing methods. Self-administered web-based tools, such as Automated Self-Administered 24-hour recall (ASA24), offer cost-effectiveness, flexibility, and rapid scaling across large areas. Mobile apps enable real-time self-tracking, barcode scanning, recipe-logging, and integration with fitness devices. However, the lack of standardized methodology and transparent food/nutrient databases limits their utility for national dietary surveillance. Image-capturing technologies, such as technology-assisted dietary assessment (TADA) and wearable cameras, enable the dynamic documentation of food consumption. However, challenges remain in scaling image analysis, linking it to nutrient data, and ensuring participant compliance. Emerging technologies, such as sensors, diet quality scores, and biomarkers, offer additional tools to objectively measure dietary intake and eating behaviors. Despite their limitations, these approaches show promise when combined with multidisciplinary strategies for precision nutrition (Moshfegh et al., 2022). The use of smartphone applications to collect dietary intake data from digital food records has gained popularity (Thompson et al., 1994). However, it is important to acknowledge that despite technological advancements, these apps are still susceptible to reactivity bias because they rely on self-reported data and may be perceived as intrusive (König et al. 2022). In the SEEM Study, the assessment of children's dietary intake employs the open-ended questionnaire traditional paper-and-pencil form method.

There are several steps to calculating dietary intake, one of which is to develop a nutrient dataset against the food items surveyed. The nutrient dataset refers to data regarding the nutritional composition of food obtained from tables or databases. It comprises the original analytical values, imputed values, calculated values, borrowed values, and assumed values. In addition to gathering these values, three methods are available for compilation: direct, indirect, and combination methods. Employing the combination method is not only cost-effective but also leads to the generation of high-quality datasets, In this method, the data is collected through both analysis and by sourcing it from scientific literature (Elmadfa et al. 2010; Greenfield and Southgate 2003). Consequently, a variety of food composition tables and datasets available at the national, regional, and international levels were leveraged to develop the nutrient dataset, as detailed here. The Food Composition Table of Pakistan (FCTP) was initially introduced in 1985 and revised and expanded in 2001 to include additional nutritional components such as zinc, iodine, vitamin A, and cholesterol-like substances. The updated FCTP contains 210 food items, including weaning recipes, supplementary dishes, and traditional recipes, commonly consumed by different age groups in Pakistan. However, it still lacks many micronutrients (Soomro et al. 2016; Bushra, 1996; Farheen, 1995; Hussain, 2001). The Indian Food Composition Tables (IFCT) and the Bangladesh Food Composition Tables provide nutritional information for over 500 food items and have complete basic nutrients and all kinds of food groups. These tables contain raw food data, and few recipes besides snacks and fast food dishes have not yet been analyzed or updated (Shaheen et al. 2013). The international food composition data source is The Food and Nutrient Database for Dietary Studies (FNDDS), which provides comprehensive nutrient information for foods reported in the National Health and Nutrition Examination Survey (NHANES). The database contained detailed nutrient profiles, portion sizes, and recipe details for these food items. This enables researchers to conduct more in-depth analyses of dietary intake, such as examining the nutrient attributes of commonly consumed foods and determining the amounts of various nutrients and food components across a wide range of food items. The international sources used for a few food items are Health Canada's Canadian Nutrient File and European Nutrient Database (Merchant and Dehghan 2006; McKillop et al. 2021; McCabe-Sellers et al., 2008),Australia and New Zealand manages databases containinof g nutrient information and food consumption > 500 foods (Authority, 2011). Interpreting food composition data requires accounting for several factors, including the description of food, reliability of the data source, quality of the data, and methods used for chemical analysis. This process involves detailed scrutiny of the data through laboratory procedures and understanding of various elements, such as the food's origin, season, processing methods, soil quality, climate conditions, and storage practices. Soil nutrient content can be altered by contaminants and the methods used to harvest crops. In addition, the timing of harvest, storage procedures, processing methods, and cooking methods can influence the nutrient content of foods. Excessive cooking can lead to the depletion of vitamins and minerals (Hinojosa-Nogueira et al. 2021; Seljak et al. 2013; Khalid et al. 2019; Korošec et al. 2013; Grande and Vincent 2020; Greenfield and Southgate 2003). Therefore, the FAO/INFOODS provides comprehensive guidelines for developing nutrient datasets, ensuring that all factors affecting data quality are considered.

Nutrition experts rely on food composition databases to assess nutrient intake and to develop tailored dietary plans. This study established a nutrient dataset for the food items examined in the Study of Environmental Enteropathy and Malnutrition(SEEM) that longitudinally followed young children of 6–24 months of age living in a rural setting in Pakistan and aimed to foster the development of sustainable food composition databases.

## 2 Materials and methods

### 2.1 Study sample and design

This study was conducted as part of an interventional research project investigating biomarkers of environmental enteropathy in malnourished children. The project, known as the Environmental Enteropathy Study (SEEM), involved the recruitment of 416 children, 51 of whom were well nourished and 365 of whom were malnourished. The children were registered within the first month of life, enrolled between 3 and 6 months of age based on their WHZ score, and were followed up on a monthly basis until 24 months. To assess children’s nutritional intake, the collection of 24-hour food recall data were collected to quantify the impact of dietary habits on children's health. 24-hours food recall form was completed on a bi-monthly basis from 6 months onwards until 24 months. The methodology for collecting 24-hour food recall data has been previously published in two papers (Iqbal et al. 2019). We selected Matiari as our study site because it has a high malnutrition burden.

In December 2015, the study protocol was approved by the Aga Khan University Ethics Review Committee (ERC, 3836-Ped-ERC-15) and the study was registered at clinicaltrials.gov (ID: NCT03588013).

### 2.2 Dietary data collection

A 24-hour food recall approach was implemented to obtain dependable information on both the consumption amount and types of food. Interviewers were responsible for questioning the mother about all the food items their children had consumed during the last 24-hours period, including breakfast, lunch, and dinner.

To facilitate data collection, we categorized food into three groups: recipes, raw food items, and processed food items for each category, and obtained detailed descriptions such as proper names, brand names, sizes, shapes, colors, major ingredients, ingredients used to prepare recipes, and whether they were peeled, unpeeled, or had inedible parts removed. In instances where the mother did not know the proper names of food products, whether branded, non-branded, or major ingredients, product information was obtained directly from the packaging. Toolkits comprising food images, household utensils such as plates, cups, and feeders, and plastic fruit models aided food measurement and ingredients to help in probing to obtain good quality 24HD data. Data over 24 h were reported in compliance with FAO/INFOODS guidelines, matching food with various nutrient data sources utilizing exact match or best match criteria (Stadlmayr et al.). The recipe data were collected separately to calculate recipe nutrient data at the household level using comprehensive cooking methods, raw ingredient descriptions, portion sizes of raw ingredients, and fat adjustments based on Eurofir recipe harmonization methods (Reinivuo et al., 2009). Further, Dietary supplement information was gathered to achieve a more comprehensive evaluation of nutrient intake (detailed questionnaire is discussed in the supplementary table).

In last of the survey the measurements were done to accurately calculate nutrient intake and recipes from the 24-hour dietary recall (24hDR) data, all food portion sizes were measured in grams. This was done using an electronic weighing balance with a sensitivity of ±1 g and a capacity of approximately 10 kg ('A digital kitchen scale 10 kg SF-400'). The measurements included each food item portion size consumed per unit utensil, recipe ingredients used in recipe as in, and further to obtain yield factor for Pakistan recipes, both pre-cooked and post-cooked recipes were measured in grams in a similar way.

### 2.3 Nutrients of concern

Key dietary components that are crucial for nutritional assessment were the focus of our study. These included total energy (kJ), total carbohydrate (g), protein (g), fat (g), saturated fat (g), monounsaturated fat (g), polyunsaturated fat (g), cholesterol (mg), iron (mg), zinc (mg), potassium (mg), calcium (mg), sodium (mg), copper (mg), magnesium (mg), vitamin A (μg RAE), vitamin E (mg), vitamin D (μg), vitamin B1 (mg), vitamin B2 (mg), vitamin B6 (mg), total folate (μg), vitamin B 3 (μg), vitamin C (mg), total dietary fiber (g), and total sugar (g). We adhered to the FAO/INFOODS standards for nutritional analysis, which ensured that the expressions, definitions, units, and denominators for the nutritional values were consistent with those in most international Food Composition Table (FCT) and Food Composition database (FCDB), except for FCTP. However, FCTP contains outdated nutrients, such as total carbohydrates and crude fiber, and uses common names for nutrients rather than the FAO/INFOODS tags. To ensure accuracy and consistency, we identified nutrients with different tag names and units, and homogenized them accordingly.

### 2.4 Selecting software for developing a nutrient dataset

Data collection, computation, analysis, and coding were performed using Excel and STATA software. The initial information gathered by the data collectors was recorded on paper and entered into separate Excel sheets for recipe and 24-hour food recall data with distinctive coding. The Langual system was used to describe each food item, and the local recipe names were translated into English. Foods were matched using the EFSA food explorer interface. STATA was utilized for analyzing and calculating recipes, adjusting for bioavailability, and approximating the nutrient intake of the children.

### 2.5 Food-matching criteria to standardize the nutrients dataset

Our goal was to determine the most suitable food match from a nutrient data source for the foods reported in the survey to obtain quality data. The FAO/INFOODS guidelines for food matching version 1.2 were used to follow the food match (Stadlmayr et al.). This match required similar names, descriptions, and nutrient values expressed in standardized definitions, manifestations, units, and denominators. In cases of no matching, rigorous and controlled methods ensured the best possible food fit.

Food matching was guided by the FAO/INFOODs standard, using the Langual™ system to describe the names of the foods reported in the survey before they were matched with other sources. The following steps were performed to ensure successful food matching (Stadlmayr et al. 2011; Charrondiere et al. 2016; Rand et al., 2011) First, food groups such as raw and processed foods were matched for the nutrient profiling system by identifying their names, descriptors, taxonomic or scientific names, water content, fat content, and key components of interest. Major ingredients in food packaging or obtained from online resources helped match branded and non-branded food products. For example, crisps commonly found in Pakistan are made primarily from corn, rice, or wheat flour, as indicated on packages. Based on these ingredients, crisps were matched to similar foods, such as rice chips and corn chips found in the nutrient composition databases.

The food components were matched according to their expression, definition, analytical methods of measurement, units, and denominators. Where fat or water content differed by 10 % or more between the Pakistani food item and its matched database food, adjustments were made by adding or subtracting the nutrients associated with water and fat. This ensured that the nutrients were standardized according to the moisture and fat levels of the original Pakistani foods (Stadlmayr et al.). This matching process allowed Pakistani foods to be profiled nutritionally and incorporated into the nutrient data system. The entire process of food matching is illustrated diagrammatically as follows.

### 2.6 Matching foods with the EFSA food explorer interface

The EFSA Food Explorer interface was utilized to code and match foods that were not available in our national and regional data sources (Bangladesh and India) (Scholz et al. 2018) This software presents food information in a standardized format, making it easier to match food items with international food composition data. The EFSA food explorer interface categorizes food into groups, such as raw primary commodities (RPCs), RPC derivatives (packaged or unpackaged food products), and composite foods (recipes), providing standardized information that facilitates matching with international food composition data. The WHO/GIFT team trained our Aga Khan University Data Analyst team to match foods using the EFSA food explorer interface (Authority 2011a) 26, 33]. We used the EFSA food explorer to match food items based on their name, cooking method, recipe ingredients, and major ingredient information available on the web and packaging. For instance, we matched biscuits branded as "Rio" by their major ingredients such as wheat flour, chocolate cream, and sugar instead of the brand name itself.

### 2.7 Matching foods with national, regional, and international food composition data sources

The food items under evaluation were cross-checked with data sources at both regional and national levels. The food composition data sources of Pakistan were prioritized to match the survyed food. However, a recent study by Sanam et al. (2016), in which mineral data of Pakistan were updated by an indirect method, found that only 20 raw Pakistani foods had their mineral compositions matched with the food surveyed under the SEEM study. Second, FCTP was used to match raw foods despite being outdated (23 years old) and not meeting the fundamental nutritional data requirements based on several FAO/INFOODS databases; the food composition table from Pakistan was still used (Soomro et al. 2016), and most raw food data were extracted for some macro and micronutrient data. However, because of its old-fashioned nutrient content in comparison to the current standard status of FAO/INFOODS, most nutrients, such as total carbohydrates and crude fiber, were excluded. In addition, lab analyses were conducted on the majority of processed (branded and non-branded) food items, such as candies, chocolates, crisps, biscuits, and nimko (a combination of roasted and fried nuts, seeds, and pulses), to determine their macronutrient content (moisture, total fat, total protein, and ash). That data is unpublished yet, we performed an analysis using the laboratory AOAC standard methods of macronutrients. The aim of examining macronutrients was to establish the exact amounts of protein and energy consumed by the children and to correlate protein quality with their growth rate. We compared the Macronutrient data and micro nutrients data from Pakistan’s FCDS and most o food items were not matched, for this reason to fill the gaps we utilized the regional food composition tables of Bangladesh (2015) (Shaheen et al. 2013) and India (2017) (Longvah et al. 2017). The Indian composition table was the primary source for nutritional data extraction, enabling a match for approximately 102 food items. The primary nutrients obtained were macronutrients, fatty acid composition, and phytate content. The regional food composition data were deemed suitable because Pakistan, India, and Bangladesh were a single country before 1947, with similar dietary and agricultural habits. In cases where the food was not matched up to the regional level, international data sources like FNDDS 2017–2018 were explored. To obtain a complete nutrient dataset, approximately 200 branded and non-branded items, mainly snacks and fast food, were matched with micronutrient data from FNDDS to 2017–2018. We also used Australian food composition databases and other online resources to perform the calculations. To follow the FAO/INFOOD units and denominator guidelines, we recalculated the energy and total carbohydrates using a different formula for the available carbohydrates. Additionally, we calculated energy by applying factors from available carbohydrates, proteins, dietary fiber, and fat.

### 2.8 The food was matched to the original nutritional label sources

Nutritional labels serve as a standardized format to provide consumers with information regarding the nutritional content of all types of food. Typically, these labels list the serving size, calories, total fat, saturated fat, cholesterol, sodium, total carbohydrates, crude fiber, sugars, and protein content of the food. In several countries, including the United States, Canada, and the European Union, the inclusion of nutritional labels in packaged foods is a legal requirement. These labels are usually located on the back or sides of the packaging and are intended to assist consumers in making informed choices regarding their food purchases. During our analysis of unpublished powdered milk data, we ensured that the values were within the range of ± 5 gm/100 gm.

When other data sources were not available, the label values were considered appropriate. We linked packaged food, including infant formula powder milk, cerelac products, and tea whiteners, with the original sources. In total, we matched 12 different brands of infant formula powder milk, three brands of cerelac dry instant baby food, and four brands of tea whitener to collect nutrient data from their nutritional labels. We matched 19 different food products with their respective nutritional labels; however, the nutrient labels proved to be insufficient because they lacked some of the essential nutrients we needed. As a result, to acquire the missing data, we applied the previously mentioned matching criteria using the U.S. database (U.S. Department of Agriculture 2022).

### 2.9 Calculating recipes for unmatched foods

**Fig. 1:**
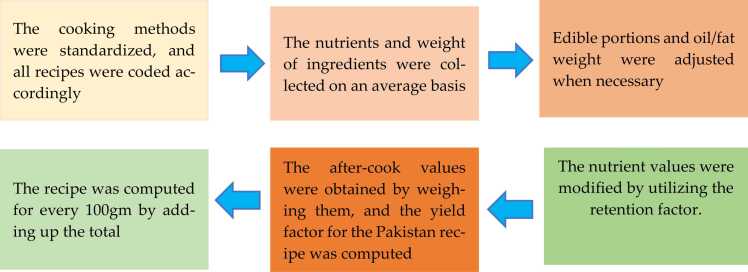
A flowchart depicting the process of creating nutritional composition data for a recipe is illustrated.

Matching food recipes is a crucial aspect of food analysis as it constitutes a significant portion of our dietary intake. Because each household prepares food differently, the number of ingredients used, the weight of primary ingredients, and yield factors vary, resulting in a significant variation in nutrient data in food recipes. The recipe data collected from our survey did not align with all sources of the food composition databases. Therefore, we calculated the nutrient values of the recipes to include them in our nutrient dataset [28]. Various methods are available in the literature for calculating recipe quantities, including the EuroFIR method, the FAO/INFOODS approach, the British method, the method employed by the European Prospective Investigation into Cancer and Nutrition (EPIC), and the USDA method (Hartmann et al. 2008; Vásquez-Caicedo et al., 2008) Schakel et al. found that a comparison of analytical and calculated values of mixed dishes by the USDA's Human Nutrition Information Service showed a difference in nutrient content of less than 10 %. This indicates that rigorous calculations can serve as valid alternatives to chemical analyses. Therefore, instead of using costly chemical analyses for each recipe, it would be beneficial to use a more cost-effective method for recipe nutrient analysis. In this study, the EuroFIR and FAO/INFOOD methods were employed, as shown in Fig. 1 [37].

**Fig. 2:**
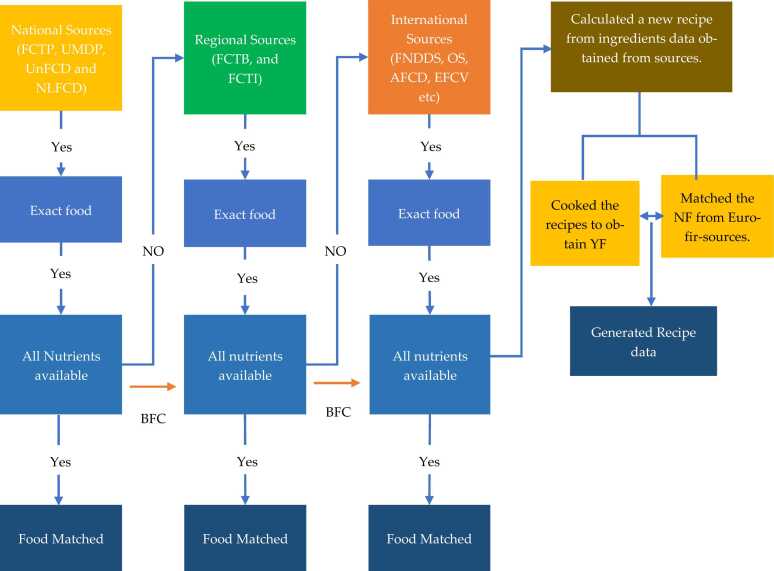
Showing a visual representation, using a flowchart, to illustrate the sequential process of matching reported foods with corresponding food matches gathered from various sources; UnFCD = Unpublished food composition data, NLFCD = Nutrients label food composition data, Estimated food composition values, BFCD = Borrowing food.

**Fig. 3:**
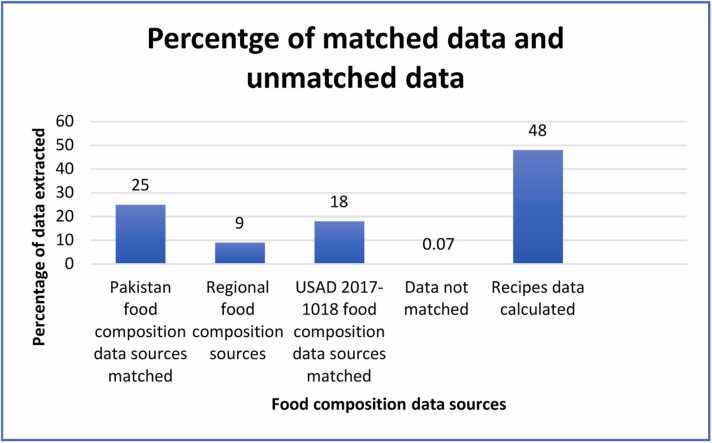
Graph of percentage matched and unmatched national, regional, and international sources.

We surveyed more than 1000 recipes and identified 162 methods. These methods often use the same ingredients in different amounts or have minor variations in the same quantities. For example, a buffalo milk recipe is cooked using one method but with different quantities of ingredients (buffalo milk: minimum 15 % and maximum 90 %; sugar: minimum 5 % and maximum 15 %; and water adjusted accordingly). Based on these variations, we created 17 different standardized composite recipes for the calculation. The composite recipes were prepared by averaging the weight of the same ingredients in the recipes, with a difference of ± 10 % for liquid foods, such as tea and boiled milk. For curry-based recipes or others, the weight of the major ingredients that significantly contribute to the nutrient content was used for averaging to make composite recipes. For example, one chicken curry sample was divided into variations based on the ingredients used. One variation included chicken curry cooked with onion, tomatoes, simple spices, and oil. The quantities of all the ingredients were averaged and combined into one composite recipe. Another variation included chicken curry with onion, tomatoes, and potatoes, and the ingredients were similarly averaged into a second composite recipe. Similarly, potato curry was cooked using different ingredients. One variation contained onion and tomato, whereas the second variation contained either onion or tomato with plain or mixed spices, or oil and ghee. This logic was applied to all other types of recipes to make n=604 composite recipes with n=162 different methods.

#### 2.9.1 Adjusting for weight changes during cooking

The variation in cooking recipes is influenced by factors such as cooking method, time, and age of the intended consumers. Recipes for children are typically altered at the household level based on the child's age, ranging from six to 24 months. At around six months of age, children primarily consume soft foods, and their eating habits evolve as they grow older and start eating what their parents prepare. This study is novel because there is a lack of information on the yield factors for recipes specific to Pakistan. The variation in recipe data from home to regional levels makes it challenging to compare. The recipe data included in this nutritional dataset were obtained from a survey that gathered information on over 162 recipes with varying cooking processes to determine yield factors. The yield factor is the weight that remains after food preparation, processing, or other treatments, and can fluctuate due to moisture or fat/solid losses or gains. The best method for estimating the yield factor is to weigh the total raw ingredients in their edible, ready-to-cook state, prepare the recipe, and then weigh the finished dish in its ready-to-serve state (FAO/INFOODS, 2012b). The yield factor (YF), which is the weight change in foods or recipes due to cooking, can be determined using the formula provided by Bognar (2002).

Yield factor = recipe uncooked value/recipe after cooking value.

Calculating the yield factor for household-level-based recipes is not feasible; hence, only recipes with similar cooking methods were selected for the yield factor calculations. For example, the most commonly prepared chicken curry variety from all households was chosen to calculate the yield factor, which was then applied to all other chicken curry varieties with similar ingredients. This approach was adopted because the yield factor data for Pakistan are not yet available. The data generated from this study could be beneficial for the creation of a Pakistan food composition table in the future, as presented in the results.

#### 2.9.2 Adjustment for nutrient changes during cooking

To overcome the lack of nutrient information for composite recipes, we estimated it using nutrient retention factors, which reflect the changes in nutrient content during cooking. The U.S. database defines true retention as the ratio of nutrients present in raw food to those in cooked food. As analytical data for cooked recipes are often unavailable, many public and commercial databases use these retention factors to calculate the nutritional value of cooked recipes. The calculated nutrient values accounted for the nutrients that were lost during the food preparation process while still being present in the food. Eurofir and FAO/INFOODS guidelines offer three different processing methods: moist heat, dry heat, and fat/oil cooking. The sub-cooking processes were categorized according to the food groups, as different groups retained nutrients at varying ratios. Our survey recipe data were matched to the exact retention factor at the ingredient level. In Pakistan, recipes typically involve two or three subcooking processes; therefore, we considered multiple RFs [28]. To correct for nutrient loss from raw food ingredients to cooking, we used the retention factor, whereas the yield factor was used to correct for loss of fat and water. The calculations were performed using Eurofir guidelines and checked for Fat, Moisture, Sodium, and Potassium within the given standard ranges.

### 2.10 Estimated calculated values

The common methods used to estimate nutrient values include using values from a similar food or various preparations of the same food, additional ingredients in the same food, home recipes, or commercial product formulations, information from nutrient labels of commercial food products, or assuming a zero value. For our study, we utilized the first two methods, specifically relying on nutrient values from another food within the same genus or family of foods and assuming a zero value.

We used the total estimated calculation to determine the phytate content of 176 food items (43 %) that could not be matched with any available sources. This method estimated the phytate content based on food group matching. For example, the phytate level of chocolate determines the phytate content of chocolate candy. Although these biscuits, chocolates, and candies had varying nutrient profiles, we assigned them the same phytate contents. We estimated the phytate content of all types of plain and cream-filled biscuits, cakes, crisps, rusks, chocolates, and candies, regardless of whether they contained cumin seeds or not, by using their primary raw to processed source ingredients without applying any factors. We applied the phytate content of finger fries to fried potatoes, obtained data for plain biscuits from other sources, and used it for all types of biscuits. Phytate is mostly present in plant-based foods, such as nuts, seeds, and grains, and cannot be detected in animal-based foods. Therefore, we assumed a phytate content of zero for pure compounds, such as sugar, or foods derived from major animal sources, such as sugar candies, packaged juices, and jellies.

## 3 Results

With a systematic and standardized approach to food matching, 89 % of the foods reported in the 24-hour dietary recall interviews were successfully matched with either an exact or equivalent food name derived from a reliable database out of them is mostly raw food items used as ingredients in recipes and ready to eat in raw form, such as fruits. Additionally, we utilized recipe calculations to match foods that were not present in the database but were composed of ingredients obtained from high-quality data sources such as national and regional databases. Table 1 Nutrient data sources that contributed to matching the nutritional information for 383 food items, including ready-to-eat and baby food items, as well as 162 calculated recipes. In our experience, the Food Explorer Interface was highly effective in correctly matching all 383 food items and uniformly arranging their codes. The FNDDS 2017–2018 database matched the nutrient content of 57 % of the food items reported in the survey (n=225). The food items are mostly processed, and the recipes predominantly include fast food items such as sandwiches, burger turnovers, and similar dishes. Additionally, the nutrients extracted were primarily micronutrients for all items, with macronutrients only included for recipes that were not collected in the dietary data. These recipes are consumed by children outside their homes. We were unable to collect data from these external sources; therefore, we generalized and matched them using international food sources. The original analytical values for macronutrients were the second most commonly used Food Composition Database (FCD) for food, matching 54 % of the total n=208 items. These values are primarily used for processed foods, such as snacks, biscuits, crisps, and candies chocolates. In the local culture, it is common for children to consume these foods with milk tea for breakfast. Regional databases provided approximately 26 % of the data, whereas our own food composition data for Pakistan covered 21 % of the food data for different nutrients. National and regional sources were used for most raw food items, such as ingredients in recipes and fruits consumed in raw form, such as mangoes and bananas. Nutrient data for recipe ingredients were used to calculate the recipe's nutritional content considering nutrient loss and yield factors. Additionally, the Food Composition Table for Pakistan (FCTP) was selected to obtain nutrients, such as protein, ash, fat, moisture, iron, zinc, calcium, phosphorus, vitamin A, beta-carotene, thiamine, riboflavin, and niacin. From the updated mineral data for Pakistan, only 20 food items were matched to obtain complete minerals. Missing nutrient information and raw food items that did not match national sources were supplemented with data from the Indian and Bangladeshi food composition databases.

**Table 1:** Contributions of percentages and food numbers from different data sources to the nutritional dataset.

**Nutrients Data Source**	**Number of Foods**	**Contribution to data source (%)**
Updated minerals data of Pakistan (UMDP)	20	5.22
Food composition table of Pakistan 2001 (FCTP)	82	21.41
Indian Food composition table 2017 (IFCT)	103	26.89
Food composition table of Bangladesh 2001 (FCTB)	12	3.13
Food and nutrition database (FNDDS 2017–2018)	222	57.96
Lab analytical data	208	54.31
EFSA Food Explorer	383	100.00
Other sources	45	11.75
Data not matched	1	0.26
**Recipe Formation**		
Raw ingredients	162	100.00
Cooked ingredients available	0	0.00
Cooked nutrients unavailable	1606	100.00
EFSA Food Explorer	1606	100.00

Because the food consumption survey included a wide range of foods, recipes were formulated for n=162 of the reported foods with different cooking methods, but the total composite recipes were n=604, which were calculated, with the majority accounting for various additions to standard recipes through EuroFIR calculations using data from national and regional food composition tables.

The distribution of the food composition sources from national, regional, and international levels, along with recipe data, is illustrated in figure-3. The majority of the food composition data (25 %) were obtained from national sources, followed by International USAD food sources and regional sources. Recipe data accounted for the largest portion (47 %) of the data. Approximately 3.4 % of the data were obtained from other local online sources and nutritional fact labels. The EFSA Food Explorer was used to match food items with codes and names, which facilitated the identification of corresponding regional and international data sources. However, we were unable to find any data on "Kum," a green-colored fruit resembling dragon fruit, despite searching various online sources. As a result, we decided to remove it from the list because it was only consumed by a single child (once a day).

## 4 Discussion

This study presents a methodology to synergistically utilize several sources of national food composition data, including the Pakistan food composition table and updated information on minerals in Pakistan. Furthermore, we acquired laboratory analytical values specifically for the analysis of macronutrients in snack foods, such as crisps, biscuits, cakes, candies, chocolates, syrups, and other similar items, to develop a new or better database for the Pakistani population where undernutrition is most prevalent.

The food composition table of Pakistan encompasses macro- and micronutrients, with a few exceptions, from our nutrient dataset (n=82, 21.4 % of food items). The collection consisted of 82 food items, comprising individual ingredients and fruits. In order to revise the mineral data for Pakistan, we consulted a scientific study conducted by Sanam et al. in 2016 However, this study only covered a limited number of cereals, fruits, vegetables, pulses, and lentils (n = 20, 2.5 %) for minerals such as Ca, Fe, Zn, Mg, Na, K, Cu, and P. Previously, we investigated the macronutrients in high-fat snack foods in an unpublished study using a similar methodology to obtain lab analytical values for other high-sugar snack foods. By merging the data from both sources, we achieved a match of 54.3 % (n = 208) for macronutrients.

About 55.7 % of the matched data percentage had missing values, which we acquired from various regional and international data sources, such as the Indian food composition table (Longvah et al. 2017) Bangladesh food composition table, (Shaheen et al. 2013) and other online sources. For recipe ingredients and fruits, we utilized regional sources to complete the missing values, with the majority of food items matched using the Indian food composition table (n = 103, 26 %).

After utilizing regional sources to fill in the missing values, we relied on the U.S food composition data to finalize the data. This high-quality data precisely matched our food items (n = 222, 57.96 %). For the micronutrient dataset of complete snacks, fast foods, and single-ingredient foods, we used the FNDDS 2017–2018. Other sources, such as the Australian food composition database and nutrition fact labels for infant powder milk, tea whiteners, liquid milk packaged, cerelac, etc., were used to match the remaining food items (n = 45, 11.75 %). Pakistan has a rich and diverse food culture that draws influence from its geography, history, and cultural traditions. A country's cuisine is a fusion of various regional and ethnic cooking styles. Our findings indicate that individuals living in the Matirai Sindh area have significant amounts of sugar, salt, and oil/fat in their recipes. Additionally, recipes meant for children in Pakistan differ significantly from those in other countries. Although our cuisine shares some similarities with Indian culture, the Indian food composition tables do not contain recipe data. Therefore, it is crucial to compile all recipes to accurately represent our cuisine. By analyzing 206 recipes for variations in ingredient proportions and portion sizes and 162 recipes for different ingredients and their respective percentages, we were able to cover approximately 46 % of the recipe data. This database proved to be crucial in identifying culturally suitable foods for the Pakistani population, as it contains a wide range of foods from Pakistan, India, Bangladesh, and the U.S database. We also recalculated the energy and total available carbohydrates from the combined nutrient data of high-quality DBs. The study utilized the Food Explorer Interface, which offers several advantages for matching foods. By incorporating the LanguaL™ thesaurus, the interface helped eliminate any ambiguity in the food descriptions. Additionally, the EuroFIR thesaurus provides a clear description of the food components in proper definitions, expressions, and units. We also followed FAO data check guidelines to ensure data accuracy.

We compared identical foods from different sources that were used in at least 15 % of our dataset. Although the majority of food composition data were consistent between the FCTP and IFCT databases, we noticed differences between the two databases. For every nutrient, we observed distinct and frequent variations between FCTP 2001 and IFCT 2017. However, it is worth noting that most of the differences were within the range of 10–50 %.

At the national level, it is important to mention that our food composition table is not up-to-date, and even if we incorporate updated mineral data, there may still be discrepancies. Our analysis revealed that the content of Fe and Zn in foods increased over time, whereas other nutrients experienced fluctuations. To compare foods extracted from both FNDDS and another dataset that primarily contained the same food items, we utilized lab analysis. We observed notable differences in fat and protein contents, ranging from 20 % to 30 %, in addition to a difference of 10–20 % in water content. Although we made every effort to minimize errors, there were some limitations beyond our control. We were unable to evaluate the micronutrient content because the lab-analyzed values were not available. Second, because phytate data were not available for most foods, it was estimated to adjust the absorbance of Ca, Fe, and Zn in the human body. Lastly, variability in the nutrient content of foods due to country-specific factors, such as climate, planting conditions, light, temperature, and soil characteristics, as well as variations in analytical methods and differences in supplementation policies between countries, might have contributed to our findings.

While this method effectively prevented missing values for the components of interest, additional research is needed to validate its accuracy for most of the foods surveyed. This can be accomplished by comparing the nutrient estimates obtained using this method with those obtained through chemical analyses.

## 5 Conclusions

By implementing the standardized stepwise approach of the combination method, we successfully generated a comprehensive nutrient dataset that aligns with the food items identified in a nutrition survey conducted in Matiari Sindh, Pakistan. This accomplishment is particularly noteworthy given that Pakistan depends heavily on its food. This research highlights the gap in the development of new food composition tables and helps assess the nutrients of populations of different age groups living in high-malnutrition settings. With no such effort made over the years, this database could be utilized to inform numerous studies focusing on the role of malnutrition, especially in children where growth faltering leads to irreversible damage to social and mental wellbeing. This cost-effective solution will guide other researchers, who might require developing a nutrient dataset in areas where reliable FCTs/FCDBs are not available.

## Ethical approval and consent to participate

Aga Khan University Hospital's ethical review committee approved the study in 2015 with ERC number 3836-Ped-ERC-15

## Funding information

This study was funded by the 10.13039/100000865Bill and Melinda Gates Foundation (AA: OPP1138727, SRM: OPP1144149. The funding agency had no role in the design of the study; collection, analysis, and interpretation of data; or in writing this manuscript.

## Author statement

The authors confirm that this work is original and has not received prior publication, nor is it under consideration for publication elsewhere. The authors have read and approved the final manuscript and agree to be accountable for all aspects of the work in ensuring that questions related to the accuracy or integrity of any part of the work are appropriately investigated and resolved.

## CRediT authorship contribution statement

**Zehra Jamil:** Writing – review & editing, Supervision, Formal analysis. **Sanam Iram Soomro:** Writing – original draft, Validation, Methodology, Formal analysis, Data curation. **sheraz ahmed:** Writing – review & editing, Supervision, Project administration. **Najma Memon:** Validation, Supervision, Resources, Methodology, Investigation, Conceptualization. **Ghulam Raza:** Formal analysis. **Khaliq Qureshi:** Resources, Project administration. **Asad Ali:** Supervision, Project administration, Funding acquisition. **Sadaf Jakhro:** Project administration. **Imran Ahmed Chauhadry:** Supervision, Formal analysis. **Fayaz Umrani:** Supervision, Resources, Project administration. **Muhammad Sajid:** Formal analysis, Data curation.

## Declaration of Competing Interest

The authors declare that they have no known competing financial interests or personal relationships that could have appeared to influence the work reported in this paper.

## Data Availability

Data will be made available on request.
