# Supplementary material for: Nutrient dataset development via FAO/INFOODS approach for infant nutritional survey in rural Matiari, Pakistan
Source: J Food Compost Anal. 2024 Sep;133:106471. doi: 10.1016/j.jfca.2024.106471 (PMC11287758; doi:10.1016/j.jfca.2024.106471)
Supplement: Supplementary file 1 — Supplementary material [file mmc1.docx]

**Supplementary table:** The questionnaire of 24-hour food recall survey under SEEM study with complete description.

| S/No: | Question | Description |
| --- | --- | --- |
| 01 | Child ID | Entered the Study Child’s unique ID number here. |
| 02 | Today’s Date | Formated DD/MMM/YY |
| 03 | Study Researcher/  Nutritionist/Fieldworker ID | Entered the Study Researcher / Nutritionist / Fieldworker’s unique ID number here. |
| 04 | Line number | This should be filled in as 1, 2, 3, etc., to enumerate each line of data |
| 05 | Food number | This should be sequentially numbered for each food item, recipe or breast milk feed, etc. |
| 06 | Meal | Identified each feeding episode/meal/snack separately. All foods/drinks that are offered together should get the same number. |
| 07 | Home | Where was the food consumed? Write “1” if it was consumed at Home and “0” if it was elsewhere. |
| 08 | Time | Recorded the time of the feed/offering of food or liquid in HH:MM in 24 hour time cycle. Our goal was to get relative times; we do not expect exact times here. |
| 09 | Recipe description | Named the recipe; Most common recipe name were used to write for better understanding |
| 10 | Recipe code | Recipes were coded with unique code |
| 11 | Food Item: description | Described the food item sufficiently to allow identification of proper code. |
| 12 | Food Item: Code | Entered the six digit code designated for this food.. |
| 13 | (R/C):Raw/Cooked | Wrote “1” if the item was raw and “2” if it was cooked |
| 14 | Food served: Portion Size description | Described what was served to the child in proportion |
| 15 | Food served: amount (in g) | Amount of portion were measured into gram by using electronic balance and were wrote on questioner |
| 16 | Food leftover: Portion size description | For more accuracy, we have asked the left portion size also to remind them again. |
| 17 | Food left over amount (in g) | Amount of portion were measured into gram by using electronic balance and were wrote on questioner |
| 18 | How many times was the child nursed during the daytime: | Wrote down the number of times the child was put to the breast for feeding during the day (sunrise to sunset) in the last 24 hours |
| 19 | How many times was the child nursed during the night: | Wrote down the number of times the child was put to the breaks for feeding during the nighttime (sunset to sunrise) in the last 24 hours. |
| 20 | Total times nursed: | Add the number in question 18 & 19 to get the total times the baby was nursed in the last 24 hours. |
| 21 | Comments | Relevant details from interviewer or coder after the interview was completed such as special food offered to the child due celebration or other festivities. Ideally, interviewer should conduct a recall interview on the day that is typical but if such instances, please note that in this section. |
| 22 | Is this form collected for secondary recall? | This question asks if the form was collected for secondary recall. If it is collected for secondary recall, ensure it was done within 2-7 days since the actual first recall. |
| 23 | Supplements | In this questionnaire, the data of the supplements were also collected, in addition, the intervention of supplements such as AchaMum and RUTF was administered to those children who were in different stages of malnutrition (Intermediate or greater). |
